# The Benefit of Mental Skills Training on Performance and Stress Response in Military Personnel

**DOI:** 10.3389/fpsyg.2019.02964

**Published:** 2020-01-14

**Authors:** Andrew E. Jensen, Jake R. Bernards, Jason T. Jameson, Douglas C. Johnson, Karen R. Kelly

**Affiliations:** Naval Health Research Center, San Diego, CA, United States

**Keywords:** resiliency, kinetic environments, stress, military training, mindfulness training

## Abstract

Mental skills training (MST) has been suggested to reduce stress in civilian and athletic populations, however, whether these techniques and practices transfer to a military population are unknown. Therefore, the purpose of this study was to evaluate two MST programs against a baseline condition, training-as-usual (TAU), during an intense, active-duty, military training environment. Two hundred and three Marines enrolled in the United States Marine Corps’ Basic Reconnaissance Course participated in this effort (*n* = 203; age = 22.7 ± 3.3 years; height = 178 ± 6.35 cm; weight = 97.7 ± 8.3 kg; Mean ± SD). Each Marine was assigned to one of three groups, Mindfulness-Based Mind Fitness Training (MMFT), General Mental Skills Training (GMST), or TAU. Operational and cognitive performance measures, as well as, physiological metrics were obtained across three training phases (phase 1–3). Furthermore, phase 3 was sub-divided into pre-ambush, ambush and post-ambush time points. Significant group × time interactions were found for the total number of errors committed on the sustained attention response task (*p* = 0.004); as well as, plasma cortisol (*p* < 0.0001) and insulin-like growth factor-1 (IGF-1; *p* < 0.0001). There were mixed results between groups on operational performance tasks with the MST groups tending to perform better than TAU the more time participants had with MST instruction. During ambush, the differences among groups were especially pronounced for measures of information processing that one would expect MST to enhance: coordinates recall, plot time, and plot accuracy (*p* < 0.001), with improvements ranging from 24.7 to 87.9% for the MST conditions when compared to TAU. These data demonstrate that independent of the specific type of MST program, the fundamental characteristics of stress regulation embedded within each MST program may enhance performance and cognitive function during time of heightened stress.

## Introduction

Persistent demands, such as those experienced during active duty military training and combat, can lead to lapses of attention and ultimately result in decreases in cognitive performance (King et al., 2006; Heaton et al., 2014). Moreover, exposure to combat stress is associated with declines in several domains vital to military operational success, such as reaction time, sustained attention, and impulse control (Lieberman et al., 2005; Schreckengaust et al., 2014; Vrijkotte et al., 2016). In the extreme, poor adaptation to combat stress is associated with impaired cognitive function, subtle neurologic compromise, neuroendocrine dysregulation, low levels of unit cohesion, and psychosocial support (King et al., 2006; Stanley et al., 2011). Given the negative consequences of stress, it is vital to train military personnel to handle not only the physical rigors of combat but the psychological as well.

Normally, the strength of the stress response, both physical and psychological, is positively correlated with the strength of the cumulative stimuli. However, emerging evidence suggests that negative responses may be attenuated through mindfulness-based training or, in general, mental skills training (MST) (Matousek et al., 2010). To do so, MST cultivates capabilities that provide the basis for successful learning and performance and promotes adaptive responses to acute and chronic stressors (Day and Livingstone, 2001; Jha et al., 2010; Fjorback et al., 2011; Stanley et al., 2011; Leonard et al., 2013; Botha et al., 2015). In fact, MST has also been shown to blunt the physiological stress response, limit cognitive decline, and reduce the amount of perceived stress (Birrer and Morgan, 2010; Matousek et al., 2010; Jha et al., 2015). Indeed, Leonard et al. (2013) demonstrated that in incarcerated youth, where there is a high level of stress, MST mitigated the decrements in attentional task performance, as compared to a control group (Leonard et al., 2013). Furthermore, Jha et al. (2010) reported increases in working memory capacity and attention in a military cohort that received MST. While these results are promising, the majority of research has been conducted in a civilian or an athletic population (Matousek et al., 2010; Leonard et al., 2013; Botha et al., 2015). And while an athlete may face the stress of a critical game or match (Mellalieu et al., 2009), military personnel are consistently exposed to a motley crew of stressors that can include sleep loss, inadequate nutrition, heavy physical training, and time away from families (King et al., 2006).

Similar to physical training, some types of MST (such as body-based arousal control) can elicit adaptations to psychological stress, thereby improving performance (Thelwell and Greenlees, 2003; Fjorback et al., 2011; Leonard et al., 2013). Moreover, the addition of MST has been shown to help limit the body’s physiological response to stress (Matousek et al., 2010), and thus may provide important protective physical effects. The importance of this possibility cannot be overstated: as it stands, continuous or sustained combat operations may strain a Warfighter’s capacity to maintain physical health and optimal performance because of intense physical effort, which results in inflammation, muscle damage, soreness, and fatigue (Knapik and Reynolds, 1997; Kraemer and Szivak, 2012; Szivak and Kraemer, 2015). Evidence suggests that the inflammatory effects of muscle damage and fatigue after strenuous physical work (i.e., regular combat maneuvers) will negatively affect the Warfighter’s physical and psychological performance in subsequent battle situations (Kraemer and Ratamess, 2005; Linnamo et al., 2005; Kersey et al., 2012; Szivak and Kraemer, 2015). In addition to the adverse inflammatory effects of military operations, there is a growing body of research indicating that the growth hormone (GH)/insulin-like growth factor (IGF)-1 axis acts as a potent mediator of physical and mental performance (Duclos et al., 2007; Giovannini et al., 2008). Indeed, growth hormone and IGF-1 act via independent and dependent mechanisms that help promote musculoskeletal growth and integrity, and improve body composition, exercise capacity, and other vital measures of health and performance (Duclos et al., 2007; Gregory et al., 2013; Schoenfeld, 2013). The influence of these hormones can either hinder or enhance/maintain the operational and performance and effectiveness of individuals (Nindl, 2009), so it is important that a method for regulating these hormones be established.

The ever present physiological and psychological demands of active duty training and combat can lead to decrements in attention, which can ultimately result in emotional dysregulation and drops in cognitive and physical performance (Nindl et al., 2002, 2007; Heaton et al., 2014; Vrijkotte et al., 2016). Importantly, performance in these critical cognitive domains can improve through training, at least in a civilian population, but whether these beneficial effects hold in a military population facing a very different set of challenges remains an understudied question. Thus, the purpose of this study is 2-fold. The primary aim is to determine if implementing MST in an intense military training course can improve cognitive and operational performance through attenuation of the stress response. Second to this, two different MST programs were evaluated to determine if delivery of MST can enhance cognitive performance and mitigate the stress response. These programs provide a way to assess contrasting approaches to the delivery of MST: one was focused primarily on teaching mindfulness-based techniques, the other was inspired mainly by techniques drawn from the domain of sports psychology. In any case, regardless of the type of MST delivery, we anticipated that performance would improve more in groups that received MST as compared to those who received training as usual (TAU).

## Materials and Methods

### Subjects

Two hundred and three active duty, male Marines currently enrolled in the Basic Reconnaissance Course (BRC) at the School of Infantry-West, Camp Pendleton, CA. Participants were briefed and had an opportunity to volunteer. All Marines who participated in this study underwent BRC training independent of this research effort. This study was approved by the Institutional Review Board at Naval Health Research Center and adhered to Department of the Navy human research protection policies (Protocol NHRC.2012.0019). All participants gave their free and informed written consent.

### Basic Reconnaissance Course Training Paradigm

The training paradigm of BRC is known for its extreme difficulty, high-intensity, and fast-paced training environment in which students train up to 16 h a day to operate behind enemy lines. The current program of instruction for BRC is 12 weeks in length and encompasses three distinct training phases, each characterized by a distinct training emphasis. The phases are broken down as follows (an overview of training can be found in Table 1):

**TABLE 1:** Overview of Marine Corps’ Basic Reconnaissance Course Training Paradigm.

**Phase**	**Baseline**	**1**				**2**			**3**				
**Week**	**0**	**1**	**2**	**3**	**4**	**5**	**6**	**7**	**8**	**9**	**10**	**11**	**12**

**MST Administration**				**X**	**X**	**X**	**X**	**X**	**X**	**X**	**X**		
**Description of Training**	**Individual and Special Reconnaissance Skills:** land navigation, combat conditioning, knot tying, rope management, supporting arms, calling for fire, and M18A1 Anti-personnel Mine.					**Amphibious Operations and Communications:** nautical navigation, amphibious reports, scouts swimming techniques, and communications security.			**Patrolling Operations:** clandestine movement, enemy contact drills, use of observation and photographic devices, and correctly reporting enemy activities.				

*MST: mental skills training. X denotes MST was provided and participants engaged in MST.*

• Phase 1: Individual training skills with an emphasis on physical strength and endurance. Training and training evaluations consist of land-based and water-based events that are timed and/or scored. Land-based events include timed runs, hikes with load, obstacle courses, high-intensity physical training, and land navigation. Water-based events occur in a pool and in the open ocean and events include high-intensity water exercises, distance swims (with and without fins), and water survival training. In addition, Marines complete classroom instruction focused on individual reconnaissance requirements.
• Phase 2: Amphibious operations training with an emphasis on the skills required to conduct maritime missions. Training and evaluations during this phase occur primarily in the open ocean environment. Marines are required to swim at a pace of 15 min per 500 yards over a total distance of 2000 yards in the open ocean with a full combat load; and to demonstrate mastery of amphibious reconnaissance skills, such as boat operations and nautical navigation from over the horizon.
• Phase 3: Patrolling operations continues on the path of the prior two phases and integrates them. During this phase, Marines are required to exhibit a mastery of the skills taught in phases 1 and 2 and to demonstrate their proficiency in an extended patrol operation in which Marines conduct a mock full-mission profile.

### Mental Skills Training Supplements

#### General Mental Skills Training

The General Mental Skills Training (GMST) program is derived from sports psychology principles and related practices existing in a similar intensity training environment, Naval Special Warfare Basic Underwater Demolition/SEAL school. Instruction was provided by the same instructor for all students. All students were provided the same training. Collectively, these practices are claimed to cultivate resilience during stressful conditions encountered during training and missions. Specifically, the primary abilities included:

• Goals and Commitment: The ability to set long and short-term goals that are intended to result in greater focus, commitment, and motivation through planning;
• Arousal Control: The ability to recognize stress symptoms and employ techniques (e.g., breath control) to reduce maladaptive reactions to stress;
• Imagery: The use of mental imagery to visualize carrying out a particular task to realize some desired outcome;
• Positive self-talk: The ability to generate an “inner monologue” containing constructive and positive characterizations of a given situation with the goal of regulating negative thoughts, feelings, and behavior during difficult times;
• Focus/Concentration: The ability to resist distractions that may arise during task performance through the use of task-specific cues.

#### Mindfulness-Based Mind Fitness Training

Mindfulness-based Mind Fitness Training (MMFT) is an MST condition that has been empirically evaluated in a sample of Marine reservists (Jha et al., 2010). MMFT adapts mindfulness skills training for a military audience. Instruction was provided by the same instructor for all students. All students were provided the same training. MMFT incorporates group instruction, integrating practice into the training regimen, and applying each skill into stressful missions with the ultimate goal of enhancing cognitive control, emotional regulation, working memory, and working memory capacity. The aims of these techniques include enhancing:

• Cognitive control: a family of attention-related regulatory processes needed to ensure that information processing is in accord with long- and short-term goals;
• Emotion regulation: a regulatory process involved in initiation or altering effective experiences and expression;
• Working memory capacity: the capacity to selectively maintain and manipulate goal-relevant information without getting distracted by irrelevant information over short intervals.

### Measures of Operational Performance

While operational measures were pre-existing assessments of the course performance, and were not developed specifically for evaluating mental skill, the measures were evaluated to determine if MST could have an impact. All performance variables were collected during one of three training phases. Phase 1 performance metrics included: the assessment of a hike, Reconnaissance Physical Assessment Test (RPAT), physical fitness test (PFT), land navigation, and the phase 1 test. Phase 2 encompassed the amphibious skills test and a final average score. Finally, phase 3 operational performance testing included a hike, patrol 1, 2, and 3, and a communications test. Each skills test/assessment are graded on a sliding scale, with the maximum score being 100 points.

### Measures of Cognitive Skill

In addition to operational performance metrics, multiple validated and operationally relevant cognitive tests were administered in a classroom setting during phase 2 (week 5) and phase 3 (week 11 and 12). Testing included: the sustained attention response task (SART) test, a date/time recall test, a coordinates recall test, a plot accuracy/time test, a facial recognition accuracy/time test, and “Kim’s game.” All but SART were designed specifically for this study. A brief description of each metric is provided below.

#### SART (Sustained Attention)

A neuropsychological test in which subjects are presented with a constant string of single digit numbers flashing one-at-a-time on a computer screen. Each number is presented for about one second. The subject is instructed to respond to every number by pressing a button, except for one designated “no-go” trial. For example, the number 3 is selected as the “no-go” trial and the subject will press the button for every number that appears except for the number 3. Due to the rhythmic and repetitive nature of the task, along with the relatively long and unpredictable intervals between the “no-go” trials, subjects are challenged to remain attentive despite the urge to respond robotically. Thus, in order to perform well, subjects must maintain attentiveness to their responses, so that, following the display of a number, they are able to override the urge and dominant motor response and substitute it with the antagonistic response. This task presents an inherent speed-accuracy trade off (Manly et al., 2000). Measurements stemming from SART included average reaction time, errors of commission, standard deviation (SD) reaction time, and total errors.

#### Date and Time Recall

Measure of subject’s ability to accurately recall the date/time group they were shown earlier during the ambush hike.

#### Coordinates Recall

Measure of subject’s ability to accurately recall the eight-digit grid coordinate they were shown earlier during the ambush hike.

#### Plot Accuracy and Time

Measure the accuracy and time to recall and plot the eight-digit grid coordinate on a map (subjects must have correctly performed the “coordinates recall” task in order to succeed in this task).

#### Facial Recognition Accuracy and Time

Subjects are given 15 s to study the pictures of four High Value Targets (HVTs) 24 h prior to this test. Four sets of four faces were shown, and one face from each set was an HVT. During the ambush hike, subjects are asked to correctly identify the profile view of each of the four HVTs. In addition to accuracy, time to respond is also measured.

#### Kim’s Game

Subjects are presented with ten operationally relevant items and given 1 min to memorize them. Subjects are tested on their ability to recall all ten items approximately 45 min later during the ambush hike.

### Measures of the Physiological Stress Response

Plasma samples were collected via venipuncture at four separate time points including a baseline measurement and three phase 3 time points; pre-ambush, ambush, and post-ambush. Collection included 1–10 mL of whole blood samples obtained via venipuncture of the antecubital vein using aseptic technique, with the subjects seated in an upright position. Blood was collected in tubes coated with ethylenediaminetetraacetic acid (Becton, Dickinson and Company, Franklin Lakes, NJ, United States). The tubes were centrifuged at 3,000 rpm for 15 min; plasma was then aliquoted into individually labeled Eppendorf tubes (Eppendorf North America, Hauppauge, NY, United States) and frozen and stored at −80°C for future analysis. The plasma samples were batch analyzed in duplicate for insulin-like growth factor and cortisol (IGF-1 and cortisol Enzo Life Science, Farmingdale, NY, United States), and epinephrine (Rocky Mountain Diagnostics, Colorado Springs, CO, United States).

### Study Design

Each platoon of recruited Marines was randomly assigned to one of three MST groups to be embedded into the 12-week BRC: TAU (*n* = 91), GMST (*n* = 47), or MMFT (*n* = 65). BRC training was divided into three distinct phases: phase 1 (weeks 1–4), phase 2 (weeks 5–7), and phase 3 (weeks 8–12). For the purpose of this investigation, phase 3 was then further divided into three time points surrounding the most stressful portion of training–the ambush. The subdivision of phase 3 includes pre-ambush, ambush, and post-ambush. Pre-ambush represents 24-hour prior to ambush, ambush represents immediately following the completion of the ambush training drill, and post-ambush represents a period 24-hour following the completion of the ambush training drill. Formal MST coursework occurred during weeks 3–10 of the BRC training cycle; TAU groups were given “free-time” during the structured MST instruction. All assessments took place at the School of Infantry (SOI) – West at Camp Pendleton. Assessment during the ambush training week took place in the field; all other assessments were carried out in a classroom setting.

### Statistical Analysis

Data were analyzed using R (v. 3.5.1, R Foundation for Statistical Computing, Vienna, Austria). A mixed effects analysis of variance (ANOVA), 4 (Time) × 3 (Group), was fit to each collected variable. For variables collected at a single time point, a one-way ANOVA comparing differences across groups was conducted. Prior to analysis, the physiological biomarkers, cortisol, IGF-1, and epinephrine were log transformed to reduce inter-subject variability. Additionally, as a measure of effect size, Hedges’ *g* (a standardized mean difference, similar to Cohen’s *d*, but adjusted for smaller sample sizes) was calculated for individual paired comparisons to provide an estimate of the magnitude of any differences between the MST groups and TAU. When a decrease in score signified an improvement in performance, scales were reversed to allow a positive effect size to always indicate improved performance. All statistical analyses were evaluated at an α-level of 0.05 (corrected, when appropriate, for multiple *post hoc* comparisons); data are reported as mean ± standard deviation.

## Results

### Subjects

Two hundred and three Marines participated in this effort (age = 22.7 ± 3.3 years, height = 178 ± 6.35 cm, weight = 97.7 ± 8.3 kg, mean ± sd). The Marines that participated had 0–4 years of combat deployment experience: 76.5% (0 years), 10.5% (1 years), 12.5% (2 years), and 0.1% (4 years) and there were no baseline differences in annual physical fitness tests between the groups.

### Measures of Operational Performance

Descriptive statistics for all pre-existing course training variables can be found in Table 2.

**TABLE 2:** Performance metrics during Marine Corps’ Basic Reconnaissance Course.

	**TAU**	**GMST**	**MMFT**
	***n* = 91**	***n* = 47**	***n* = 65**
***Phase 1* (*weeks 1–4*)**			
Phase 1 Hike	85.615.3	92.76.8^**,†††^	75.812.8^***^
RPAT^a^	0.970.18	1.00	1.00
PFT	93.384.52	94.265.74	92.886.87
Land Navigation Avg	87.238.08	86.6511.2	87.1510.11
Phase 1 Test	93.384.65	87.975.39^***^	88.925.62^***^
***Phase 2 (weeks 5–7)***			
Amphibious Skills	89.66.05	89.564.48	90.774.31
Final Average	81.685.92	91.994.3^***^	81.495.53^†††^
***Phase 3 (weeks 8–12)***			
Phase 3 Hike	90.30.7	95.61.2^***^	93.41.0^∗^
Patrol 1 Score	87.195.87	86.715.27	85.922.99
Patrol 2 Score	83.55.6	86.124.61	85.044.25
Patrol 3 Score	87.084.35	89.533.83	87.923.27
Communication Test	91.70.7	88.41.3^∗^	85.91.1^***^
Patrol Skills	90.985.21	90.594.51	88.234.25

*MMFT: Mindfulness-Based Mind Fitness Training. GMST: general mental skills training. TAU: training-as-usual. RPAT: reconnaissance physical assessment test. PFT: physical fitness test. ^∗^Significant adjusted contrast comparing MST to TAU. ^†^Significant adjusted contrast comparing GMST to MMFT, ^∗^*p* < 0.05, ^∗∗^*p* < 0.01, ^∗∗∗^*p* < 0.001, ^†^*p* < 0.05, ^††^*p* < 0.01, ^†††^*p* < 0.001. ^*a*^Pass/Fail Scoring: Recorded as 0 = Fail and 1 = Pass. All data are presented as Mean ± SD.*

#### Phase 1

Results from the one-way ANOVA yielded statistically significant differences among the three groups for phase 1 hike (*F* = 28.9, *p* < 0.0001) and phase 1 test (*F* = 12.2, *p* = 0.0001). Adjusted *post hoc* comparisons indicated higher performance scores for GMST when compared to TAU (*F* = 2.9, *p* = 0.004) and MMFT (*F* = 29.5, *p* < 0.0001) and TAU when compared to MMFT (*F* = 20.11, *p* < 0.0001) during the phase 1 hike. During the phase 1 test, TAU outperformed both GMST (*F* = 4.1, *p* < 0.001) and MMFT (*F* = 3.8, *p* < 0.001), but there was no evidence of any reliable difference between the MST groups. There was no evidence to indicate any reliable differences among groups for the RPAT, PFT, or Land Navigation tests (*p* > 0.05).

#### Phase 2

Results from the one-way ANOVA yielded statistically significant differences among the three groups for final average (*F* = 25.5, *p* < 0.0001). Adjusted *post hoc* comparisons indicated significantly higher performance for individuals participating in the GMST condition when compared to the MMFT (*F* = 6.2, *p* < 0.0001) and TAU (*F* = 6.8, *p* < 0.0001) groups; comparison of MMFT and TAU failed to reach statistical significance (*p* > 0.05). There were no differences between groups for the amphibious skills test (*p* > 0.05).

#### Phase 3

Results from the one-way ANOVA yielded significant differences at the *p* < 0.05 level between groups for phase 3 hike (*F* = 8.5, *p* = 0.0004) and communication test (*F* = 10.3, *p* < 0.0001). Adjusted *post hoc* comparisons indicated higher performance scores for GMST (*F* = 3.8, *p* < 0.0001) and MMFT (*F* = 2.6, *p* = 0.01) relative to TAU during the phase 3 hike. However, TAU outperformed both GMST (*F* = 2.2, *p* = 0.04) and MMFT (*F* = 4.4, *p* < 0.0001) during the communication test, and there was no supporting statistical evidence of differences between the MST groups (*p* > 0.05). Finally, for the patrol test, there was no evidence of reliable differences among the groups (*p* > 0.05).

### Measures of Cognitive Function

**FIGURE 1:**
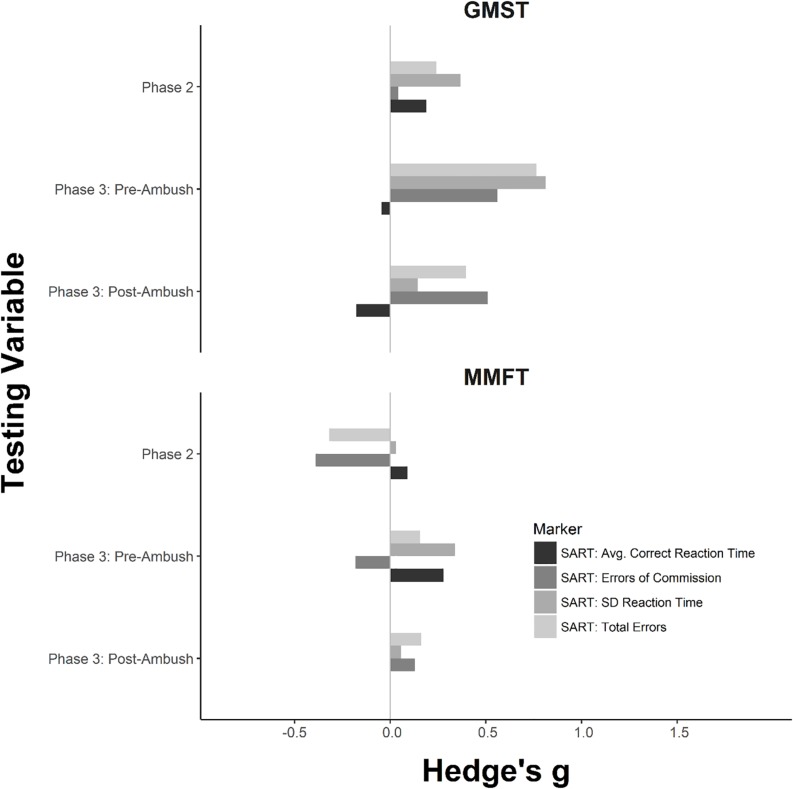
Sustained Attention to Response Task effect size comparison of group differences between General Mental Skills Training (GMST), Mindfulness-Based Mind Fitness Training (MMFT), and Training-as-Usual (TAU). The horizontal axis represents the Hedge’s *g* for each observation. The vertical axis represents the measured variable. Zero indicates no difference between the MST group and TAU. Larger positive effect sizes, indicated by a bar to the right beyond 0, indicate desired differences for MMFT and GMST when compared to TAU. Negative effect sizes, indicated by a bar to the left beyond 0, indicate an undesired effect when compared to TAU.

**FIGURE 2:**
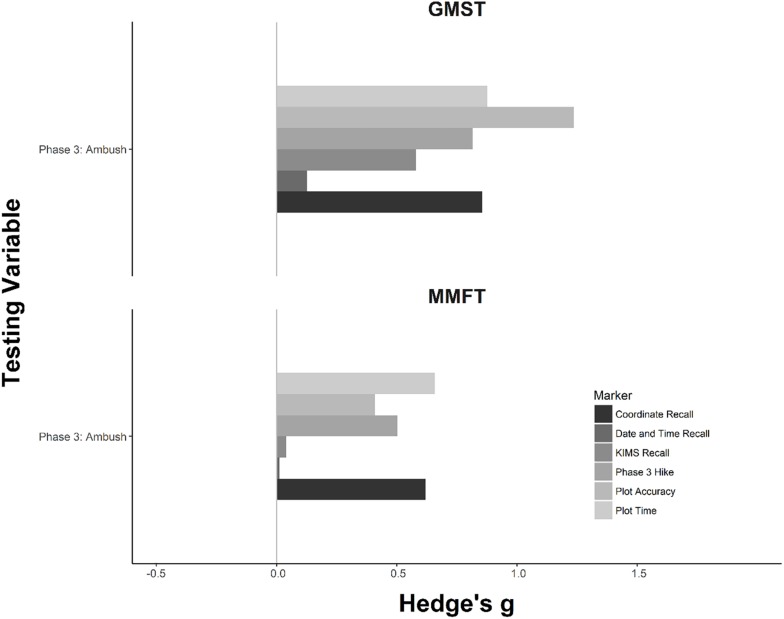
Effect size comparisons of group differences between GMST, MMFT, and TAU during the highly stressful Ambush training phase. The horizontal axis represents the Hedge’s *g* for each observation. The vertical axis represents the measured metric. Zero indicates no difference between the MST group and TAU. Larger positive effect sizes, indicated by a bar to the right beyond 0, indicate desired differences for MMFT and GMST when compared to TAU. Negative effect sizes, indicated by a bar to the left beyond 0, indicate an undesired effect when compared to TAU.

Effect sizes for the cognitive measures can be found in Figures 1, 2. For each SART metric, a mixed effects ANOVA was conducted. There was a significant interaction between the effects of group × time on total errors (*F* = 19.0, *p* = 0.003). Simple effects analyses revealed significant differences among groups during the pre-ambush time point (*F* = 3.3, *p* = 0.03). Adjusted simple contrasts revealed statistically fewer total errors for GMST when compared to TAU (*F* = 2.6, *p* = 0.03). Comparisons of MMFT to TAU and MMFT to GMST failed to reach statistical significance following the correction (*p* > 0.05).

A one-way ANOVA was computed for each remaining cognitive variable collected during the highly stressful ambush training period to determine any group differences in performance. ANOVAs determined significant differences among groups for coordinates recall (*F* = 6.2, *p* = 0.003), plot time (*F* = 5.9, *p* = 0.004), and plot accuracy (*F* = 8.9, *p* = 0.0003). Adjusted planned contrasts for coordinates recall revealed greater performance for GMST (*F* = 3.0, *p* = 0.01) and MMFT (*F* = 2.7, *p* = 0.01) when compared to TAU and no differences among MST conditions. Similarly, adjusted planned contrasts for plot accuracy showed higher performance for GMST when compared to TAU (*F* = 4.2, *p* = 0.0002) and MMFT (*F* = 2.4, *p* = *0.02*). Comparison of MMFT and TAU failed to reach statistical significance (*p* > 0.05). Results from the plot time planned contrasts showed significantly faster times for GMST (*F* = 2.9, *p* = 0.01) and MMFT (*F* = 2.7, *p* = 0.01) when compared to TAU. No differences were detected when comparing MST conditions (*p* > 0.05).

### Measures of the Physiological Stress Response

#### Plasma Cortisol

**FIGURE 3:**
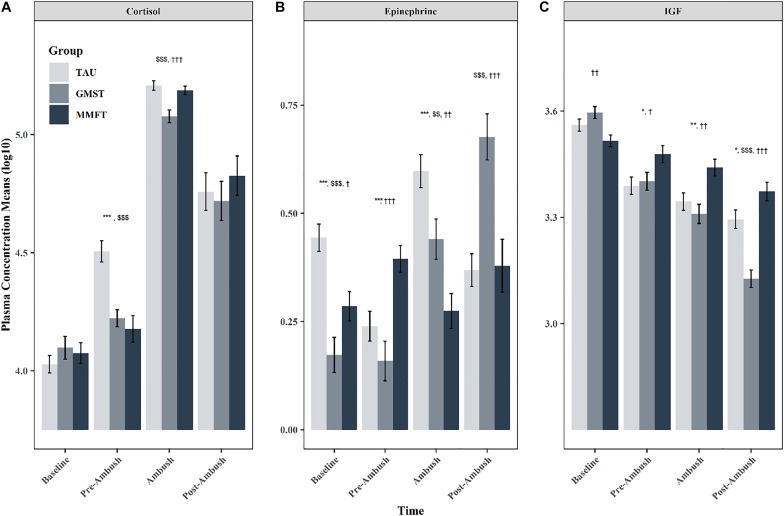
Log10 transformed means for **(A)** Cortisol, **(B)** Epinephrine, and **(C)** IGF-1 comparing the differences between GMST, MMFT, and TAU. Error bars represent one standard error above and below the mean. ^∗^indicates significant adjusted contrast comparing TAU to MMFT. ^∗^*p* < 0.05, ^∗∗^*p* < 0.01, ^∗∗∗^*p* < 0.001. ^†^indicates significant adjusted contrast comparing GMST to MMFT. ^†^*p* < 0.05, ^††^*p* < 0.01, ^†††^*p* < 0.001. ^[*dollar*]^indicates significant adjusted contrast comparing TAU to GMST. ^[*dollar*]^*p* < 0.05, ^[*dollar*][*dollar*]^*p* < 0.01, ^[*dollar*][*dollar*][*dollar*]^*p* < 0.001.

Plasma cortisol results are reported in Figure 3A. The mixed effects ANOVA revealed a statistically significant group × time interaction (*F* = 28.6, *p* < 0.0001) for cortisol concentrations. Simple effects analyses determined significant differences among groups during the pre-ambush (*F* = 14.7, *p* < 0.0001) and ambush (*F* = 10.3, *p* = 0.0001) time points. Adjusted simple contrasts for the pre-ambush time point revealed statistically lower cortisol concentrations for GMST (*F* = 4.3, *p* < 0.0001) and MMFT (*F* = 5.0, *p* < 0.0001) when compared to TAU. Comparisons of MST conditions during the pre-ambush time point showed no statistical differences (*p* > 0.05). Adjusted simple contrasts for the ambush time-point revealed statistically lower cortisol concentrations for GMST when compared to TAU (*F* = 4.3, *p* = 0.0001) and MMFT (*F* = 3.6, *p* = 0.0001), however, there were no differences between the MMFT and TAU conditions during the ambush time point (*p* > 0.05).

#### Epinephrine

Results for epinephrine are reported in Figure 3B. The mixed effects ANOVA yielded a significant group × time interaction (*F* = 91.2, *p* < 0.0001) for epinephrine concentrations. Simple effects analyses determined significant differences among groups during all four time points: baseline (*F* = 13.7, *p* < 0.0001), pre-ambush (*F* = 10.0, *p* = 0.0001), ambush (*F* = 14.8, *p* < 0.0001), and post-ambush (*F* = 11.7, *p* < 0.0001) time points. The simple contrasts analyses revealed that TAU had significantly higher epinephrine concentrations when compared to GMST (*F* = 5.1, *p* < 0.0001) and MMFT (*F* = 3.3, *p* = 0.001) at baseline. Comparison of MST groups at baseline indicated that MMFT had higher epinephrine concentrations than GMST (*F* = 2.2, *p* = 0.02). During the pre-ambush time point, MMFT had higher epinephrine concentrations when compared to GMST (*F* = 4.4, *p* < 0.0001) and TAU (*F* = 2.9, *p* = 0.007). During the ambush time point TAU exhibited the highest concentrations of epinephrine when compared to GMST (*F* = 2.7, *p* = 0.009) and MMFT (*F* = 5.4, *p* < 0.0001); furthermore, comparison of MST conditions revealed reduced concentrations of epinephrine in the MMFT group when compared to GMST group (*F* = 2.8, *p* = 0.009). Lastly, during the post-ambush phase, GMST exhibited the higher circulating concentrations when compared to MMFT (*F* = 4.1, *p* = 0.0001) and TAU (*F* = 4.3, *p* = 0.0001). No other differences were observed for epinephrine.

#### Insulin-Like Growth Factor (IGF-1)

Plasma IGF-1 results are reported in Figure 3C. The mixed effects ANOVA yielded a significant group × time interaction (*F* = 106.1, *p* < 0.0001) for IGF-1 plasma concentrations. Simple effects analyses determined significant differences among groups during all four time points: baseline (*F* = 5.3, *p* = 0.005), pre-ambush (*F* = 3.9, *p* = 0.02), ambush (*F* = 7.1, *p* = 0.001), and post-ambush (*F* = 23.7, *p* < 0.0001) time points. According to the adjusted simple contrasts, MMFT had significantly lower IGF-1 concentrations when compared to GMST (*F* = 3.2, *p* = 0.005) at baseline and significantly higher IGF-1 concentrations when compared to GMST (*F* = 2.2, *p* = 0.04) and TAU (*F* = 2.6, *p* = 0.03) during pre-ambush. During the ambush time point, MMFT continued to exhibit higher IGF-1 concentrations when compared to GMST (*F* = 3.6, *p* = 0.002) and TAU (*F* = 2.7, *p* = 0.01). The largest differences among IGF-1 occurred during the post-ambush time point where adjusted contrasts revealed significantly higher IGF-1 concentrations for MMFT when compared to GMST (*F* = 6.7, *p* < 0.0001) and TAU (*F* = 2.2, *p* = 0.03). Furthermore, during the post-ambush time point, TAU exhibited higher IGF-1 concentrations when compared to GMST (*F* = *p* < 0.0001). All other contrasts for IGF-1 failed to reach significance (*p* > 0.05).

## Discussion

The Basic Reconnaissance Course (BRC) is an advanced infantry training course in which Marines improve upon their operational skillset with a heavy emphasis on ground and amphibious reconnaissance training. Due to the volume of tactical skill that is taught, coupled to the physical rigors of military training, this advanced course is extremely demanding, both physically and mentally. Additionally, Marines are expected to be proficient in multiple weapon systems which require calling in coordinates and adjusting fire for artillery, air, and/or naval strikes. Understandably, this type of training is both highly dynamic and stressful. Thus, BRC was selected to test the efficacy of MST to enhance operational and cognitive performance. To the knowledge of the authors, this was the first study to evaluate the effect of MST on changes in stress response proteins; as well as operational and cognitive performance in an advanced military training course. The primary finding of this effort was that MST attenuates the stress response and improves performance on operationally relevant tasks as well as cognitive capabilities. Results between the two MST programs varied depending on the metric assessed and thus, the effectiveness of one program over another is not clear. However, it is clear that implementation of MST resulted in improvements in performance during high intensity training compared to TAU.

### Operational Performance

Operational performance is paramount to mission success of Marines and as such, increasing or maintaining performance during times of enhanced stress is vital to mission success. As expected, there were no differences between MST groups and TAU during operationally relevant assessments administered prior to MST (i.e., RPAT, PFT, and Land Navigation). These results suggest that all groups were of similar physical fitness and basic navigation expertise levels at entry. However, within 2 weeks of MST administration, at the end of phase 1, there were changes observed in the culminating hike between the MST and TAU groups, with GMST performing best, TAU second, and MMFT third. These findings suggest that training performance may improve in as little as 2 weeks of GMST, however, the TAU group performed better on the phase 1 test compared to the other groups, which contradicts the notion that MST can have beneficial effects in as little as 2 weeks. Furthermore, differences in cognitive function were not detectable when evaluated during week 5 (phase 2). Little is known about the specific time course for when certain effects should be observable as a result of MST. Certainly, some of these measures exhibit a closer connection to those abilities directly trained by MST (e.g., SART), and thus would be expected to exhibit clear evidence of learning, if such learning had occurred. For example, Leonard et al. (2013) found improvements in sustained attention after 3–5 weeks of MST; and Jha et al. (2010) found improvements in working memory capacity after 8 weeks. Other measures, such as some of the pre-existing course measures, maintain a more tenuous connection to abilities cultivated by MST (e.g., RPAT or the final course average), and so very little can be predicted about when these measures might show any movement in response to MST, assuming they move at all.

Phase 2 of training requires a higher cognitive load than phase 1, as water-based navigation and scout swimming are more mentally taxing due to the sight limited environment of water versus land-based training. The increased cognitive load and increased duration of MST training provided up to this point should permit adaptations of stress resilience to occur in MST groups (Stanley et al., 2011). In fact, operational performance metrics indicate that trainees who received GMST outperformed both MMFT and TAU on the phase 2 final score (Table 2).

Similar to the phase 2 performance metrics, the phase 3 performance metrics were of a higher cognitive load than that of phase 1 and encompassed the cumulative training learned over the course of BRC. Like phase 2, during the phase 3 hike GMST performed better than TAU, however, the MMFT group also performed better than TAU. Despite both MST groups performing better on the phase 3 hike, both MST groups performed significantly worse on the communications test than the TAU group. These findings were unexpected as the communications test is a cognitive stressor in which MST should enhance performance.

### Cognitive Performance

Highly stressful military training and combat simulations lead to decrements in vital cognitive domains (Lieberman et al., 2005, 2006, 2016). However, there is evidence that MST may mitigate cognitive decrements in Marines during training (Jha et al., 2010, 2015; Stanley et al., 2011). In line with Jha et al. (2010), the results from this study suggest that MST can enhance key cognitive functions. In fact, the results of this study indicate that 86% of the cognitive metrics assessed showed at least some improvement in those groups receiving MST compared to TAU.

Cognitive assessments were administered during phases 2 and 3 of BRC. Results from the phase 2 testing suggest small improvements in cognitive performance for GMST and mixed results for MMFT when compared to TAU (Figure 1). Consistent with Leonard et al. (2013), GMST displayed practical improvements in sustained attention to the magnitude of a small effect; despite this, the results failed to reach statistical significance at this time point (Hopkins, 2002). These divergent changes among MST groups through phase 2 could be explained in multiple ways. One possibility is that the mode of delivery may have had an effect. While GMST was delivered by a retired United States Marine Corps Gunnery Sergeant who previously graduated BRC, MMFT was externally sourced (outside of the military) and taught by an individual with less experience in the Marine Corps and BRC command structure. Although speculative, varying rapport between Marines and their instructor may explain the variance between the two MST groups (Roos et al., 2015).

Analysis of phase 3 pre-ambush testing illustrates that both MST groups displayed enhanced sustained attention and reaction time when compared to TAU (Figure 1). Heightened cognitive performance exhibited by MST conditions directly prior to the ambush training exercise is consistent with previous literature showing improvements in working memory capacity, attentional stability, and attentional lapses following the completion of an MST course (Jha et al., 2010, 2015; Leonard et al., 2013). Although the pre-ambush time point may have been less physically stressful, the anticipation and preparation of the ambush event itself likely increased the level of psychological stress in the Marines (True and Benway, 1992; Mellalieu et al., 2009).

Although research indicates that increased levels of stress diminish cognitive functions such as speed, attention, and executive function, the results of this study suggest that MST may mitigate stress-induced declines in cognition (Lupien et al., 2009; Gotlib and Joormann, 2010). Furthermore, others have suggested that minimizing cognitive declines is critical for successful emotion regulation which ultimately leads to enhanced stress resilience during highly stressful situations (Dienstbier, 1989; Gross, 1998, 2002; Jha et al., 2010). Consistent with these findings, evaluation of cognitive performance variables directly related to the ambush mission (i.e., coordinate recall, plot time, plot accuracy) revealed moderate to large statistically significant improvements for MST conditions when compared to TAU (Hopkins, 2002). Enhancements by MST groups remain consistent with previous literature showing improved cognitive functioning with MST (Jha et al., 2010, 2015). Notably, the degree of cognitive performance differences among conditions was largest during the most stressful BRC time points suggesting that MST skills are most effective during times of heightened stress.

### Physiological Stress Response

Improvements in the way an individual responds to a stressful situation can be monitored by physiological markers such as cortisol, IGF-1, and epinephrine. When the body is unable to respond to increased levels of stress, amplified by the combination of training stress and various outside stressors with inadequate recovery (i.e., environmental factors, sleep deprivation, diet), various circulating hormone concentrations are affected, and therefore, operational and cognitive performance perpetually deteriorates (Gleeson and Bishop, 2000). However, it is possible to maintain a limited psychosomatic control over stressors in an attempt to mitigate the harmful effects of hormonal dysfunction during times of heightened stress (Davidson et al., 2003; Robinson et al., 2003; Carlson et al., 2004, 2007; Matousek et al., 2010). Indeed, the results of this study indicate that MST training can help attenuate the physiological stress response.

Measurement of the ubiquitous stress hormone, cortisol, provides key insight into the body’s physiological response to physical and psychological stress. The results of this study indicate that all subjects’ baseline cortisol concentrations were similar; but once subjected to perceived mental stress during the pre-ambush phase, cortisol levels rose in all subject groups. While a positive correlation between cortisol and stress is expected, those in the MST groups experienced a reduced increase in circulating cortisol when compared to the TAU groups. These findings are consistent with others (Carlson et al., 2004, 2007; Matousek et al., 2010) who found that MST can mitigate the cortisol response. While cortisol levels were significantly lower in the MST groups during the pre-ambush phase, cortisol levels from all groups rose during the actual ambush and post-ambush phases, but did not differ reliably from one another. The similarity of the cortisol levels during the ambush is most likely due to the biological necessity of cortisol which during maximal physical and cognitive efforts helps promote blood glucose regulation as seen during the fight-or-flight response (Sapolsky et al., 2000). Like cortisol, epinephrine levels spike to increase metabolic rate in an attempt to prepare the body to fight or flee. However, unlike cortisol, results from this study do not indicate that MST can regulate epinephrine to any discernable amount.

Opposing the catabolic hormone cortisol, IGF-1, an anabolic peptide, acts as a marker of health and homeostasis, particularly with respect to caloric balance (Nindl, 2009; Henning et al., 2014). During times of negative caloric balance, high energy flux, and/or high stress, fight-or-flight situations, IGF-1 is decreased (Nindl et al., 2003; Rarick et al., 2007; Henning et al., 2014). Thus, if MST can attenuate the perceived stress and/or alter the energy flux, then IGF-1 levels may be sustained during BRC training. In fact, the results herein indicate that IGF-1 was increased immediately following ambush and 24-hour post-ambush in the MMFT group. Unfortunately, in the absence of carryover performance variables due to study constraints, the time course response of IGF through the ambush and post-ambush training cycles, as well as the relationship to MST and specific performance outcomes remains to be determined.

### Limitations

The relationship between MST and performance should be considered, bearing in mind various limitations of the study design due to the constraints of the BRC training schedule and access restrictions to the trainee population. First, it was not possible to control for potential instructor confounds, which opens up the possibility of bias toward the instructor to influence the effectiveness of the content. While this may be a limitation, it also may be a finding worth pursuing in a future effort: does the delivery of the material impact how an individual perceives the information? Effectiveness of the delivery, especially in a military population, is critical. If there is no “buy in” from the participants receiving MST then likely it will not will not resonate with them and thus, confound the results. Second, direct measures of practice time were not gathered, either through practice logs or semi-structured interviews. Thus, a dose-dependent response cannot be established between MST training and performance. BRC is a rigorous course and while the initial study design included practice logs, it was not practical given the environment. Furthermore, it would be difficult to discern how much independent time is required for retention of the information as well as impact on the mental and subsequent physiological response of each individual due to inter- and intra-individual responses. Given the demanding nature of the course, records maybe influenced based upon the training that was performed that day. Lastly, while highly specific and valuable to the United States Marine Corps, new measurement criteria (e.g., operational tests/assessments) provide challenges to establish validity and reliability of measurement.

## Conclusion

Despite the aforementioned limitations, the results from this study fit with a growing literature, but extend prior studies with a more ecologically valid design. Overall, these results suggest that incorporating MST into BRC training may lead to improvements in cognitive performance. Further, MST attenuates the physiological stress response and, in some instances, improves physical performance. Although practical constraints and limitations limit the strength of these conclusions, especially with respect to identifying causal mechanisms, beneficial effects for preserving or improving physical and cognitive performance during the highly stressful phases of BRC training do appear to be associated with MST, regardless of the course provided (MMFT or GMST). Currently, these data suggest that GMST is trending to be more beneficial than MMFT in operational and cognitive tasks; this may be due to the fact that the students were more receptive to a program developed within the Naval Special Warfare community. However, further work is needed to optimize the amount of MST required to elicit the changes as well as establish which aspects of MST are most relevant to the military. Nevertheless, it can be concluded that MST, in some capacity, is better than doing nothing at all and is a non-invasive method of arousal control, attention enhancement, and potential behavior modification.

## Data Availability Statement

The datasets generated for this study are available on request to the corresponding author.

## Ethics Statement

The studies involving human participants were reviewed and approved by Naval Health Research Center Institutional Review Board. The patients/participants provided their written informed consent to participate in this study.

## Author Contributions

AJ, JB, JJ, and KK analyzed and interpreted the data and wrote the manuscript. DJ designed the study and collected the data.

## Conflict of Interest

The authors declare that the research was conducted in the absence of any commercial or financial relationships that could be construed as a potential conflict of interest.
